# Prospective Safety Evaluation of a Cardiovascular Health Dietary Supplement in Adults with Prehypertension and Stage I Hypertension

**DOI:** 10.1089/acm.2018.0311

**Published:** 2019-02-15

**Authors:** Jennifer Joan Ryan, Douglas Allen Hanes, Jamie Corroon, Jan Taylor, Ryan Bradley

**Affiliations:** ^1^ Helfgott Research Institute, National University of Natural Medicine, Portland, OR.; ^2^ Family Medicine and Public Health, University of California, San Diego, La Jolla, CA.; ^3^ Australian Research Centre on Complementary and Integrative Medicine (ARCCIM), University of Technology Sydney, Ultimo, New South Wales, Australia.

**Keywords:** dietary supplements, safety, tolerability, cardiovascular health, prehypertension, hypertension

## Abstract

***Objective:*** To prospectively examine the long-term safety of a cardiovascular health dietary supplement by assessing a comprehensive set of safety measures.

***Design:*** Single-arm, open-label study.

***Location:*** National University of Natural Medicine, Portland, OR.

***Subjects:*** Thirty adults with screening blood pressure readings consistent with prehypertension or stage I hypertension.

***Intervention:*** One caplet per day of a dietary supplement for 6 months. The investigated herbal–mineral supplement contains several ingredients, most notably *Rauwolfia serpentina*.

***Outcome measures:*** Primary measures included b-type natriuretic peptide (NT-proBNP), aspartate aminotransferase (AST), alanine aminotransferase (ALT), estimated glomerular filtration rate (eGFR), electrolytes, and the Patient Health Questionnaire (PHQ-9). Exploratory measures included physical vital signs, cholesterol levels, high-sensitivity cardiac troponin-I, cystatin C, endothelin, interleukin (IL)-6, IL-17a, tumor necrosis factor-α, high-sensitivity C-reactive protein, blood counts, and the Patient Reported Outcome Measure Information System (PROMIS) Sleep Disturbance Short Form 8b.

***Results:*** NT-proBNP, AST, ALT, eGFR, sodium, calcium, magnesium, PHQ-9 score, and the majority of exploratory measures did not change. However, serum potassium increased (*p* < 0.05), systolic blood pressure decreased (*p* < 0.0001), and diastolic blood pressure decreased (*p* < 0.0001). There were no serious adverse events, but 30% of participants withdrew citing potential side effects, most commonly nasal congestion or fatigue; most participants who reported nasal congestion also reported concomitant seasonal allergies. Adherence to the supplement was 90.9%.

***Conclusions:*** The findings of this study suggest that the investigated dietary supplement is safe for long-term use in adults with prehypertension and stage I hypertension. Additional results of this study, particularly the increase in serum potassium and decreases in systolic and diastolic blood pressure, are promising and suggest that future research on this dietary supplement, or its ingredients, should further explore effects on blood pressure and biologic mechanisms of action, which may involve potassium-sparing and diuretic effects.

## Introduction

Use of dietary supplements in the United States is widespread, with more than 166 million estimated users and an estimated 85,000 products available.^1,2^ Adults with cardiovascular disease have a particularly high prevalence of use.^3,4^ An analysis of the 1999–2002 National Health and Nutrition Examination Survey (NHANES) data showed that 64% of respondents with hypertension took dietary supplements.^5^

There is a common misperception among health care professionals that dietary supplements are unregulated.^1^ However, dietary supplements *are* regulated in the United States as a special category of foods by the Food and Drug Administration (FDA) under the Dietary Supplement Health and Education Act (DHSEA).^1,2^ Regulatory agencies also exist in Canada, Europe, China, India, and Australia.^2^ In the United States, dietary supplement manufacturers are not required to conduct prospective safety studies, although they are prohibited from marketing products or using ingredients known to be unsafe.^2^ Adverse events (AEs) occur with dietary supplements, but recent estimates indicate a relatively low incidence, despite frequently voiced concerns regarding dietary supplement safety.^1,2^ Notably, less than one death per year has been attributed to use of dietary supplements over the last 15 years, compared with more than 25,000 annual deaths in the United States attributed to FDA-approved prescription drugs.^1^

However, the limited availability of formal research on dietary supplements creates a barrier to more widespread clinical use. This is particularly relevant for naturopathic physicians and other complementary medicine providers, who frequently discuss the safety, quality, and efficacy of dietary supplements during clinical encounters.^6,7^ A lack of safety-oriented research on the tens of thousands of available dietary supplements generates both clinical and scientific limitations, as potentially safe and efficacious supplements may never be identified or described in peer-reviewed medical literature.^2^ Consequently, these limitations also represent opportunities to apply innovative early-phase study designs to assess safety and also identify potentially efficacious dietary supplements.^2,8,9^

The authors previously reported on the naturopathic treatment of hypertension, in which a specific Ayurvedic herbal–mineral formula was recommended to 50.6% of the patients.^10^ On subgroup analysis, use of the formula was associated with greater reductions in blood pressure, compared with those who had not been given the supplement (unpublished data). Herbs in the formula, including *Rauwolfia serpentina*, *Rosa vinca*, and *Convolvulus pluricaulis*, each have a long history of use in Ayurvedic management of patients with hypertension.^11–13^ Also in the formula, *Rosa centifolia* is commonly used in Ayurvedic formulas for inflammatory conditions and *Tribulus terrestris*, *Terminalia arjuna*, and *Boerhaavia diffusa* each increase diuresis.^14–17^ Expanding on the aforementioned observational findings, the authors designed the present study to (1) prospectively evaluate the Ayurvedic herbal–mineral formula based on a comprehensive set of objective safety and tolerability measures; (2) assess patient-reported outcomes during extended use of the supplement; and (3) describe adherence among adults with pre- and stage I hypertension.

## Materials and Methods

### Study design

A single-arm, open-label clinical trial designed to assess safety and tolerability by prospectively assessing changes in clinical laboratory biomarkers, validated questionnaires for depression and sleep, clinical AEs, and vital signs was implemented. Primary measures were defined *a priori* as changes in (1) sodium, potassium, calcium, magnesium, aspartate aminotransferase (AST), alanine aminotransferase (ALT), estimated glomerular filtration rate (eGFR), b-type natriuretic peptide (NT-proBNP); and (2) Patient Health Questionnaire (PHQ-9)-measured mood scores. Participants were first screened for eligibility by phone and then confirmed with an in-person clinical screening visit. Once enrolled, participants returned for a baseline visit, a 3-month midpoint visit, and a 6-month closure visit. Participants were contacted by phone between study visits. All study-related operations were conducted at the National University of Natural Medicine (NUNM). This study was approved by the Institutional Review Board at NUNM and registered at ClincalTrials.gov (NCT02452749).

### Participants and recruitment

Adults ages 18–75 years with blood pressure meeting JNC 7 criteria for prehypertension or stage I hypertension (120–139 mmHg systolic and/or 80–99 mmHg diastolic) were recruited from the Portland, OR, metropolitan area.^18^ Target enrollment was *n* = 30.

Participants with the following were excluded: resting heart rate <50 beats per minute (bpm) or <45 bpm in athletes; serum sodium, potassium, or calcium levels outside of laboratory reference ranges; elevated AST or ALT; eGFR ≤60 mL/min; PHQ-9 ≥ 10; initiation of, or changes to, prescription blood pressure-lowering medications or thyroid medications within the previous month; use of β blockers, α blockers, digoxin, levodopa, antidepressant medications, antipsychotic medications, sedatives, tranquilizers, *B. diffusa*, *C. pluricaulis*, *R. serpentina*, *R. centifolia*, *R. vinca*, *T. arjuna*, *T. terrestris*, or coral powder within the previous 2 months; or participation in another interventional research study within the previous month.

Participants were excluded if they had a history of cardiovascular disease, heart surgery, cardiac arrhythmia, pacemaker placement, abnormal electrocardiogram, abnormal echocardiogram, kidney disease, chronic liver disease, gallbladder disease, bowel disease, ulcers, alcoholism, obstructive sleep apnea, Parkinson's disease, depression, pheochromocytoma, or malignancy (within the previous 5 years); reported consumption of more than 14 alcoholic beverages per week, smoking or tobacco use, illicit or recreational drug use. Lactating or pregnant women, or women planning pregnancy during the study period, were excluded. Also, those with a known intolerance or allergy to ingredients in the study supplement.

Recruitment approaches included newspaper advertisements, online advertisements, and flyers. In addition, electronic health records of consenting patients of the NUNM Health Center were queried. Potentially eligible individuals were mailed an invitation letter and recruitment flyer on a rolling basis.

### Study intervention

The intervention, Carditone^®^, was a supplement manufactured and supplied by Ayush Herbs, Inc. (Redmond, WA) in 60 caplet bottles. Participants were asked to take 1 caplet nightly before bed. The supplement contains the following ingredients in each caplet: *R. centifolia*, *B. diffusa*, and *Dendrogyra cylindrus* (coral powder) as a proprietary blend (350 mg), magnesium aspartate (200 mg), *C. pluricaulis* (100 mg), *T. arjuna* (100 mg), *T. terrestris* (100 mg), low-reserpine *R. serpentina* (50 mg), and *R. vinca* (25 mg). The manufacturers grow the herbs in the formula on farms that they own and process them in their plant in Nagrota Bagwan, Himachal Pradesh, India. The supplement is manufactured in the United States in a Good Manufacturing Practice-certified facility. The finished supplement was analyzed for reserpine content via high-performance liquid chromatography and levels were below the lower limit of detection (<0.01%; Intertek, Champaign, IL). Lead analysis showed that levels were <0.2 ppm (Micro Quality Labs, Inc., Burbank, CA).

### Data collection

Fasting blood samples were obtained by venipuncture by a certified phlebotomist. Vital signs were measured by trained clinical research coordinators. Blood pressure and resting heart rate were measured using an Omron digital blood pressure monitor (HBP-1300) after the participant was seated for at least 5 minutes with their back supported, feet flat on the floor, and their arm supported at heart level. Three consecutive readings were taken (at least 1 minute apart) and then averaged. All questionnaires were completed by direct interview. To assess adherence, unused study supplement was returned for pill counts at the midpoint and endpoint of the trial.

#### Clinical laboratory measures

Serum samples from the screening visit were sent to Quest Diagnostics (Seattle, WA) by courier the day of collection and analyzed for sodium, potassium, calcium, AST, ALT, and eGFR to confirm eligibility; however, screening laboratory values were not used in any analyses. At subsequent visits, serum, plasma, and whole blood were shipped to Veridia Diagnostics (Round Rock, TX) on the day of collection and analyzed for sodium, potassium, calcium, magnesium, NT-proBNP, AST, ALT, eGFR, total cholesterol, low-density lipoprotein cholesterol, high-density lipoprotein cholesterol, triglycerides, high-sensitivity cardiac troponin-I, endothelin, cystatin C, high-sensitivity C-reactive protein, interleukin (IL)-6, IL-17a, tumor necrosis factor-α, white and red blood cell (RBC) count, platelet count, hemoglobin, and hematocrit.

#### Participant-reported outcomes

AEs were monitored by specific interviews during scheduled 3- and 6-month visits and by phone calls between visits. Participants were encouraged to report any AE between study visits or phone calls by contacting a clinical research coordinator or the principal investigators. A 91-item standardized monitoring questionnaire, developed according to the National Cancer Institute's Common Terminology Criteria for Adverse Events v4.0, was administered at each trial visit. Administering the form requires querying symptoms in eight systems: eyes/ears/nose/throat, gastrointestinal, neurologic/musculoskeletal, psychologic/general, cardiopulmonary, skin, genitourinary, and constitutional systems. Symptoms are graded from 0 to 5 (0 = not present; 1 = mild [no intervention required], 2 = moderate [activities of daily living limited], 3 = severe or medically significant, but not immediately life threatening [basic self-care limited or hospitalization required], 4 = life threatening, 5 = fatal). Furthermore, AEs were classified as serious or nonserious based on FDA and Federal Food Drug and Cosmetic Act definitions. AEs were considered “serious” if a participant's outcome had included the following: a life-threatening experience, inpatient hospitalization, disability or incapacity, death, a congenital anomaly or birth defect, or a medical or surgical intervention to prevent one of these outcomes.^19,20^ All other AEs were designated “nonserious.”

The 9-item Patient Health Questionnaire (PHQ-9) was used to assess changes in mood. The PHQ-9 was scored as previously described.^21^ An increase of >5 points was defined as a categorical change in mood, and thus was specifically evaluated at the 3- and 6-month visits. Changes in sleep quality were assessed with the Patient Reported Outcome Measure Information System (PROMIS) Sleep Disturbance Short Form 8b, which was scored as previously described.^22,23^

### Data analysis

Continuous measures are presented as mean and standard deviation at each time point. Changes from baseline to 3 months and from baseline to 6 months were summarized using Glass's Delta, a version of Cohen's *d* that is computed as the change in means divided by baseline standard deviation; standardized differences are referred to as Cohen's *d* in the Results section. Significance of changes from baseline was drawn from random intercept models applied to measurements at all three time points. When outcomes showed substantial skew or outliers, the authors conducted sensitivity analyses using log transformations of the data, or omitting outliers, as appropriate. As part of primary aims of the study, the proportion of participants who met several *a priori* defined safety and tolerability criteria at the midpoint and trial end visits was calculated. These safety and tolerability criteria were as follows: NT-proBNP >400 pg/mL; sodium, potassium, calcium, or magnesium ≥25% higher or 25% lower than the laboratory reference range; AST or ALT >2 times the upper laboratory reference range; eGFR decrease of ≥15 mL/min from baseline; increase in PHQ-9 score by ≥5 points (without alternative explanation); systolic hypotension (<90 mmHg); diastolic hypotension (<60 mmHg); systolic blood pressure increase of ≥20 mmHg compared with baseline; diastolic blood pressure increase of ≥10 mmHg compared with baseline; resting heart rate <50 bpm (or <45 bpm in athletes); grade 3, 4, or 5 AEs (monitored via the 91-item symptom monitoring form); or participant elected to withdraw from the study citing AEs. Statistical analyses were performed using SPSS v.20 software (IBM Corp., Armonk, NY).

## Results

### Participant characteristics

**FIG. 1.**
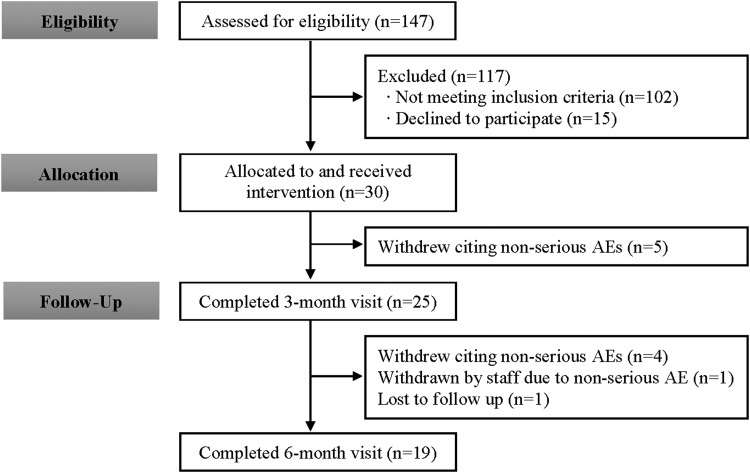
Study flow diagram. AEs, adverse events.

Baseline demographic parameters are described in Table 1. Mean systolic and diastolic blood pressure readings at baseline were consistent with JNC 7 classification of prehypertension. Flow of the study is described in Figure 1; 19 participants completed the study, 9 elected to withdraw, 1 was withdrawn by the investigators, and 1 was lost to follow-up.

**Table 1.** Baseline Characteristics of the Study Population

	*Mean ± SD or* n *(%)*
Age (years)	51.0 ± 10.4
Gender	
Male	17 (56.7)
Female	13 (43.3)
Race	
White	24 (80.0)
More than 1 race	3 (10.0)
Asian	2 (6.7)
American Indian or Alaska Native	1 (3.3)
Ethnicity	
Not Hispanic or Latino	25 (83.3)
Hispanic or Latino	5 (16.7)
Body mass index (kg/m^2^)	28.1 ± 5.4
Resting heart rate (bpm)	65.8 ± 8.2
Blood pressure	
Systolic (mmHg)	133.7 ± 10.5
Diastolic (mmHg)	80.6 ± 7.5
Taking antihypertensive medication	6 (20)

bpm, beats per minute.

### Primary measures

Significant changes in primary measures were infrequent, both from baseline to the 3-month visit and from baseline to the 6-month visit (Table 2). However, changes in potassium levels were significant both from baseline to 3 months and from baseline to 6 months (*p* < 0.05). Changes in NT-proBNP, sodium, calcium, magnesium, AST, ALT, and eGFR were nonsignificant. Primary outcome measures plus additional safety and tolerability criteria were also examined as a composite (Table 3).

**Table 2.** Primary Measures

				*Baseline to 3 months*		*Baseline to 6 months*	
*Parameter*	*BaselineMean ± SD*	*3 monthsMean ± SD*	*6 monthsMean ± SD*	*Cohen's* d	p^a^	*Cohen's* d	p^a^
NT-proBNP (pg/mL)	49.17 ± 33.46	56.17 ± 52.59	55.59 ± 42.93	0.13	0.210	0.14	0.890
Sodium (mmol/L)	141.10 ± 2.29	141.16 ± 2.32	141.53 ± 2.79	0.03	0.770	0.21	0.260
Potassium (mmol/L)	4.48 ± 0.30	4.60 ± 0.32	4.66 ± 0.23	0.53	0.040	0.56	0.019
Calcium (mg/dL)	9.45 ± 0.32	9.50 ± 0.22	9.42 ± 0.37	0.10	0.470	0.14	0.650
Magnesium (mg/dL)	2.12 ± 0.18	2.12 ± 0.18	2.13 ± 0.19	0.00	0.920	−0.03	0.940
AST (U/L)	20.63 ± 4.38	20.52 ± 5.09	22.06 ± 9.18	−0.08	0.730	0.24	0.480
ALT (U/L)	21.60 ± 8.08	21.56 ± 7.15	23.00 ± 16.77	−0.06	0.860	0.11	0.620
eGFR (mL/min/BSA)	97.27 ± 14.36	93.80 ± 14.91	90.29 ± 16.56	−0.10	0.380	−0.13	0.370
PHQ-9 (total score)	1.70 ± 1.86	2.96 ± 3.31	3.26 ± 4.24	0.54	0.053	0.53	0.078

^a^ *p*-Values calculated using mixed-model analysis, random intercept model.

ALT, alanine aminotransferase; AST, aspartate aminotransferase; BSA, body surface area; eGFR, estimated glomerular filtration rate; NT-proBNP, b-type natriuretic peptide; PHQ-9, Patient Health Questionnaire.

**Table 3.** Safety and Tolerability Criteria Composite

	*3 months*		*6 months*	
*Parameter*	*Proportion*	*Percentage*	*Proportion*	*Percentage*
NT-proBNP >400 (pg/mL)	1 of 25	4.0	0 of 19	0.0
Sodium ≥25% higher or 25% lower than the laboratory reference range	0 of 25	0.0	0 of 19	0.0
Potassium ≥25% higher or 25% lower than the laboratory reference range	0 of 25	0.0	0 of 19	0.0
Calcium ≥25% higher or 25% lower than the laboratory reference range	0 of 25	0.0	0 of 19	0.0
Magnesium ≥25% higher or 25% lower than the laboratory reference range	0 of 25	0.0	0 of 19	0.0
AST >2 times the upper laboratory reference range	0 of 25	0.0	0 of 19	0.0
ALT >2 times the upper laboratory reference range	0 of 25	0.0	2 of 19	10.5
eGFR reduction of ≥15 mL/min/BSA from baseline	1 of 25	4.0	2 of 19	10.5
Increase in PHQ-9 score by ≥5 points (without alternative explanation)	2 of 25	6.7	0 of 19	0.0
Systolic hypotension (<90 mmHg)	0 of 25	0.0	0 of 19	0.0
Diastolic hypotension (<60 mmHg)	0 of 25	0.0	1 of 19	5.3
Systolic blood pressure increase of ≥20 mmHg compared with baseline	0 of 25	0.0	0 of 19	0.0
Diastolic blood pressure increase of ≥10 mmHg compared with baseline	1 of 25	4.0	0 of 19	0.0
Resting heart rate <50 bpm (or <45 bpm in athletes)	0 of 25	0.0	1 of 19	5.3
New-onset moderate or greater severity dizziness	0 of 25	0.0	0 of 19	0.0
Grade 3, 4, or 5 adverse events	0 of 30	0.0	0 of 25	0.0
Withdrew from the study citing adverse effects	5 of 30	16.7	4 of 25	16.0
Composite	9^a^ of 30	30.0	10 of 25	40.0

^a^ At the midpoint visit, nine participants met at least one of these criteria. One participant had both elevated NT-proBNP and an increase in diastolic blood pressure of ≥10 mmHg compared with baseline. These adverse changes were attributable to a new prescription medication (gabapentin prescribed after baseline) and inadvertently doubling supplemental niacin the morning of the study visit. This individual was withdrawn as a precautionary measure. Their NT-Pro-BNP was repeated after withdrawal and had returned to within-normal limits. Their NT-proBNP data were excluded from the analysis.

ALT, alanine aminotransferase; AST, aspartate aminotransferase; BSA, body surface area; bpm, beats per minute; eGFR, estimated glomerular filtration rate; NT-proBNP, b-type natriuretic peptide; PHQ-9, Patient Health Questionnaire.

### Adverse events

No serious AEs were reported by any participant during the study. Nine participants (30%) elected to withdraw before completion citing nonserious AEs as the reason (Table 4). AEs reported by 10% or more of the entire study population included the following: lightheadedness (*n* = 9, 30%), nasal congestion (*n* = 6, 20%), feeling tired/fatigued (*n* = 6, 20%), and headache (*n* = 3, 10%).

**Table 4.** Participant Withdraws Citing Adverse Events

*Sign/symptom*	*Number of adverse events*	*Notes*
Nasal congestion	3	History of seasonal allergies noted in two of the three individuals; a third participant commented that wildfires (causing diminished local air quality) may have contributed to their symptoms
Felt tired/fatigued	3	
Sleep disturbance	2	One participant reported sleep disturbance due to nasal congestion; another participant reported insomnia and graphic dreams
Hypotension (self-report)	1	Was on concomitant antihypertensive medication
Lightheadedness, mild	1	
Headaches	1	Worsening of preexisting symptom
Feeling depressed and unmotivated	1	Most attributable to several personal adjustment issues

Twelve total signs/symptoms were reported by nine participants who elected to withdraw. One participant who reported nasal congestion (with concomitant seasonal allergies) also reported sleep disturbance. Another participant with nasal congestion (and seasonal allergies) also reported feeling tired/fatigued. The participant who reported mild light-headedness also reported worsening of preexisting headaches.

### Exploratory measures

Significant changes in exploratory measures were more frequent than in primary measures (Table 5). Decreases in systolic and diastolic blood pressure were highly significant at 3 and 6 months compared with baseline (*p* < 0.0001). Several blood measures also changed significantly, including cystatin C, RBC count, hemoglobin, and hematocrit (*p* < 0.05). However, mean values for these biomarkers remained within normal laboratory reference ranges at all time points. There were no significant changes in high-sensitivity cardiac troponin-I, endothelin, cholesterol, inflammatory markers, white blood cell and platelet counts, sleep questionnaire total score, or heart rate.

**Table 5.** Exploratory Measures

				*Baseline to 3 months*		*Baseline to 6 months*	
*Parameter*	*Baseline Mean ± SD*	*3 months Mean ± SD*	*6 months Mean ± SD*	*Cohen's* d	p^a^	*Cohen's* d	p^a^
Cardiac troponin-I (pg/mL)	1.03 ± 0.66	0.98 ± 0.63	1.09 ± 0.59	−0.10	0.550	0.04	0.600
Endothelin (pg/mL)	2.16 ± 0.55	2.25 ± 0.66	2.05 ± 0.37	0.11	0.520	−0.20	0.390
Total cholesterol (mg/dL)	198.63 ± 37.34	199.84 ± 36.14	203.29 ± 32.27	0.05	0.610	−0.14	0.580
LDL (mg/dL)	129.30 ± 35.64	131.64 ± 35.51	129.00 ± 31.88	0.06	0.480	−0.21	0.140
HDL (mg/dL)	55.27 ± 12.74	54.52 ± 13.64	56.88 ± 15.26	0.03	0.720	0.06	0.600
Triglycerides (mg/dL)	117.43 ± 53.64	115.92 ± 57.41	124.82 ± 73.83	−0.01	0.750	−0.13	0.680
hs-CRP (mg/L)	1.46 ± 1.62	1.42 ± 1.30	2.80 ± 5.38	−0.05	0.940	0.60	0.140
Interleukin-6 (pg/mL)	0.94 ± 0.59	1.13 ± 0.81	0.88 ± 0.34	0.26	0.170	−0.13	0.900
Interleukin-17A (pg/mL)	0.47 ± 0.29	0.73 ± 0.96	0.48 ± 0.26	0.73	0.160	−0.31	0.720
Tumor necrosis factor-α (pg/mL)	2.05 ± 0.75	1.98 ± 0.65	1.92 ± 0.56	−0.16	0.360	−0.17	0.390
Cystatin C (mg/L)	0.82 ± 0.14	0.87 ± 0.14	0.93 ± 0.16	0.24	0.010	0.40	0.001
White blood cell count (thousand/μL)	5.58 ± 1.08	5.50 ± 1.24	5.18 ± 0.88	0.04	0.810	−0.20	0.270
Red blood cell count (million/μL)	4.79 ± 0.39	4.77 ± 0.42	4.64 ± 0.36	−0.06	0.540	−0.24	0.030
Hemoglobin (g/dL)	14.35 ± 0.79	14.19 ± 0.94	14.04 ± 0.80	−0.21	0.100	−0.34	0.010
Hematocrit (%)	43.88 ± 2.72	43.39 ± 2.75	42.67 ± 2.61	−0.18	0.180	−0.34	0.010
Platelet count (thousand/μL)	223.17 ± 58.33	213.04 ± 62.79	190.58 ± 46.66	−0.01	0.890	−0.21	0.120
PROMIS sleep disturbance total score	16.43 ± 5.00	17.84 ± 7.08	16.11 ± 5.58	0.36	0.160	−0.08	0.950
Heart rate (bpm)	65.80 ± 8.21	63.68 ± 21.08	58.11 ± 6.75	−0.19	0.645	−0.69	0.134
Systolic blood pressure (mmHg)	133.73 ± 10.47	119.28 ± 11.75	120.11 ± 8.70	−1.59	<0.0001	−1.58	<0.0001
Diastolic blood pressure (mmHg)	80.60 ± 7.54	71.56 ± 7.59	71.16 ± 7.42	−1.22	<0.0001	−1.19	<0.0001

^a^ *p*-Values calculated using mixed-model analysis, random intercept model.

bpm, beats per minute; HDL, high-density lipoprotein; hs-CRP, high-sensitivity C-reactive protein; LDL, low-density lipoprotein; PROMIS, Patient Reported Outcome Measure Information System.

### Study supplement adherence

A pill count of unused study supplement indicated that adherence throughout the study was 90.9% ± 11.6%.

## Discussion

This trial rigorously collected long-term safety and tolerability data on a dietary supplement using a semipragmatic model. The supplement evaluated has been sold in the United States for 25 years and is recommended by naturopathic and complementary medicine providers for cardiovascular health. Although many of the findings were unremarkable, several findings stand out as clinically meaningful, including a lack of serious AEs and a lack of meaningful change in many biomarkers, including NT-proBNP, three of four electrolytes, liver enzymes, and eGFR. The lack of change in these measures suggests safety and a lack of harm to major organ systems, including the kidney, liver, and myocardium. Significant decreases in systolic and diastolic blood pressure were also clinically meaningful. The modest but significant increase in serum potassium was also clinically interesting and potentially related to modulation of blood pressure.

The decreases in blood pressure were anticipated based on historic use of the supplement, prior research findings, and previously reported mechanisms of action of the ingredients.^10^ These results support previously observed findings that suggested that use of the formula was associated with clinically meaningful blood pressure reductions when the formula was used as part of a comprehensive naturopathic treatment plan to address hypertension. Regarding potential mechanisms of action of the formula studied, *R. serpentina* is rich in alkaloids that exhibit multiple pharmacologic actions, including a vasodilatory effect and alteration of the balance between the sympathetic and parasympathetic nervous systems.^24^ *R. vinca* contains alkaloids that act as α-adrenergic receptor antagonists.^12^ *T. terrestris*, *T. arjuna*, and *B. diffusa* each increases diuresis.^15–17^ *T. terrestris* and *B. diffusa* also each inhibit angiotensin-converting enzyme (ACE) activity.^17,25^

The observed increase in potassium was unexpected, since modulation of serum potassium was not a previously known clinical effect of the formula. However, the increase in potassium is consistent with previously reported preclinical data on ingredients in the formula; *T. arjuna* and *T. terrestris* have each been shown to increase potassium, along with diuresis, in animal models.^15,16^ Furthermore, the increase in serum potassium could have been related to potential inhibition of ACE activity by *T. terrestris* and *B. diffusa*.^17,25^ It is well documented that agents that inhibit ACE have the downstream effect of reducing renal potassium excretion.^26^ However, this study was not designed to assess ACE activity.

Interestingly, mean cystatin C increased throughout the study, yet remained within the normal laboratory reference range. Cystatin C is a biomarker of glomerular filtration and unlike eGFR, which is an *estimate* of glomerular filtration rate, cystatin C is independent from serum creatinine, age, gender, race, and muscle mass.^27,28^ Since cystatin C is highly sensitive to change in glomerular filtration rate, the slight increase in serum cystatin C is plausibly related to increased urine output (diuresis), if the supplement did indeed increase diuresis.^28^ However, this study was not designed to assess urine output.

This analysis also demonstrated small but significant decreases in RBC count, hemoglobin, and hematocrit over the course of the study. Epidemiologic data collected from racially diverse populations in multiple studies demonstrated that RBC count, hemoglobin, and hematocrit are *each* highly correlated with both systolic and diastolic blood pressure.^29–32^ Hematocrit in particular is the prevailing determinant of blood viscosity and as a result plays a role in long-term modulation of blood pressure.^29,33^ It is plausible that the slight decreases in RBC count, hemoglobin, and hematocrit observed after 6 months are related to the decreases in systolic and diastolic blood pressure demonstrated in the first 3 months of the study.

Although there were no serious AEs, 30% of the study sample elected to withdraw due to nonserious AEs. The nonserious AEs reported by 10% or more of the entire study population were not surprising given previously published clinical data. The mild and typically transient light-headedness reported was most likely related to the observed change in systolic and diastolic blood pressure. Many agents, particularly antihypertensives, can cause dizziness.^34^ Nasal congestion is a well-known effects of *R. serpentina*.^11^ A long-term study on the effect of *R. serpentina* extract in hypertensive individuals published in 1956 reported nasal congestion in 30% of participants.^35^ In the present study, 20% of participants reported nasal congestion. All but one individual who reported nasal congestion also reported preexisting seasonal allergies, suggesting that seasonal allergies may be a contraindication for some individuals. *R. serpentina* is also known to cause fatigue and *T. arjuna* has been reported to cause headache.^24,36^

This study has both strengths and limitations. Strengths include successful implementation of a robust experimental design, devised to collect long-term and comprehensive data on an herbal–mineral formula not previously evaluated in a prospective clinical study. An additional strength was excellent adherence by the participants as demonstrated by pill count data. While not including a control group may be perceived as a limitation, an early-phase study does not always require an untreated group according to Aickin, considering that the real purpose of early-phase research is to inform and influence future research effort.^8^ Carefully designed single-group studies can suffice depending on the response of participants.^8^ A clinically relevant limitation of the study was evaluating one dosage of the dietary supplement. The authors investigated a dosage of one caplet per day, but health care providers who recommend the supplement will sometimes suggest a dosage regimen of up to four caplets per day, based on the clinical presentation of each patient. The high rate of withdrawal from the study was also an important limitation, especially in a study with a relatively small number of participants. The high rate of withdrawal was partly a reflection of the study design, which collected data from participants over a 6-month period, a lengthy time frame for a dietary supplement study.

A randomized, placebo-controlled trial for the treatment of hypertension is justified to confirm and expand on the clinically meaningful decreases in systolic and diastolic blood pressure observed in the present study. It is plausible that the changes in potassium and blood pressure are related, and thus, future research should explore if the mechanisms involve potassium-sparing and diuretic effects. In the meantime, the results presented here greatly inform the clinical community about what to expect from the use of this dietary supplement in practice, including rates of clinically relevant AEs, reasons for potential limitations in adherence, the expected magnitude of blood pressure decreases at a typical starting dosage, and cautions of potential interactions with other agents, including prescription potassium-sparing diuretics.

## Conclusions

The findings of this study suggest that the investigated dietary supplement is safe for long-term use in adults with prehypertension and stage I hypertension. Additional results of this study, particularly the decreases in systolic and diastolic blood pressure, and the potentially related increase in serum potassium, can also serve to inform future clinical research endeavors that explore efficacy and mechanisms of action of the investigated dietary supplement or its ingredients. A follow-up study, that includes a control arm and tests additional dosages of the supplement, is justified to expand on the findings of this study.
